# *DeepVelo*: Single-cell transcriptomic deep velocity field learning with neural ordinary differential equations

**DOI:** 10.1126/sciadv.abq3745

**Published:** 2022-11-30

**Authors:** Zhanlin Chen, William C. King, Aheyon Hwang, Mark Gerstein, Jing Zhang

**Affiliations:** ^1^ Department of Statistics and Data Science, Yale University, New Haven, CT 06520, USA.; ^2^ Healthcare and Life Sciences, Microsoft, Redmond, WA 98052, USA.; ^3^ Mathematical, Computational, and Systems Biology, University of California, Irvine, Irvine, CA 92697, USA.; ^4^ Department of Molecular Biophysics and Biochemistry, Yale University, New Haven, CT 06520, USA.; ^5^ Department of Computer Science, Yale University, New Haven, CT 06520, USA.; ^6^ Department of Computer Science, University of California, Irvine, Irvine, CA 92697, USA.

## Abstract

Recent advances in single-cell sequencing technologies have provided unprecedented opportunities to measure the gene expression profile and RNA velocity of individual cells. However, modeling transcriptional dynamics is computationally challenging because of the high-dimensional, sparse nature of the single-cell gene expression measurements and the nonlinear regulatory relationships. Here, we present *DeepVelo*, a neural network–based ordinary differential equation that can model complex transcriptome dynamics by describing continuous-time gene expression changes within individual cells. We apply *DeepVelo* to public datasets from different sequencing platforms to (i) formulate transcriptome dynamics on different time scales, (ii) measure the instability of cell states, and (iii) identify developmental driver genes via perturbation analysis. Benchmarking against the state-of-the-art methods shows that *DeepVelo* can learn a more accurate representation of the velocity field. Furthermore, our perturbation studies reveal that single-cell dynamical systems could exhibit chaotic properties. In summary, *DeepVelo* allows data-driven discoveries of differential equations that delineate single-cell transcriptome dynamics.

## Teaser

*DeepVelo* uses neural ordinary differential equations to learn single-cell transcriptome dynamics.

## INTRODUCTION

Recent advances in single-cell RNA sequencing (scRNA-seq) allow us to simultaneously measure gene expression profiles of individual cells, opening new avenues for investigating cellular development at an unprecedented resolution (*1*–*6*). A major goal in scRNA-seq is to study cell development and differentiation (*7*, *8*). In particular, dissecting the regulatory cascade underlying cell state transitions is crucial for understanding key biological processes such as embryonic development, tissue regeneration, and oncogenesis (*9*–*12*). However, most scRNA-seq experiments only capture a gene expression snapshot, making it difficult to continuously track the transcriptomic profile of the same cells over time.

Several methods, such as *Monocle* and *Palantir*, can uncover broad developmental trends by modeling discrete cell state transitions using pseudotime (*13*, *14*). Despite their success, these methods were not designed to model continuous-time transcriptome dynamics within individual cells (*15*). More recently, RNA velocity was proposed to estimate instantaneous transcriptome changes via the ratio of spliced versus unspliced transcripts (*16*). While promising in various complex tissues, RNA velocity can only predict future cell states on the time scale of hours (*17*–*19*). To mitigate the instantaneity, linear ordinary differential equations (ODEs) and sparse approximation–based methods were developed to predict the continuous evolution of cell states over a longer period of time (*20*, *21*). However, transcriptional regulation is a precisely coordinated biological process that requires complex coordination of numerous regulators, e.g., nonlinear interactions among obligate heterodimer transcription factors (TFs) (*22*, *23*). Therefore, these linear models may underestimate the regulatory complexity and fail to capture the nonlinearity of gene expression dynamics.

Inspired by recent developments in neural ODEs and data-driven dynamical systems (*24*, *25*), we present *DeepVelo*, a neural network–based framework to formulate the dynamics underlying scRNA-seq experiments and to overcome the aforementioned challenges. Specifically, *DeepVelo* trains a variational autoencoder (VAE) to predict the rate of change in gene expression (e.g., RNA velocity) from a measured transcriptomic profile. Then, we embed the VAE into an ODE to model continuous changes in gene expression within an individual cell over time. As a result, our deep-learning architecture can model complex nonlinear gene interactions in a regulatory cascade, reduce the dimensionality of the gene expression dynamics, and learn to denoise velocity fields in scRNA-seq. Most importantly, by piecing together cells from different developmental stages, *DeepVelo* can make accurate cell state predictions farther into the future. Distinct from existing methods, *DeepVelo* learns neural differential equations to describe continuous-time single-cell gene expression dynamics.

To illustrate the robustness and general validity of our approach, we performed a proof-of-concept case study on mouse pancreatic endocrinogenesis (*26*). Then, we applied *DeepVelo* to decipher the gene expression dynamics behind the developing mouse brain, specifically in the dentate gyrus and neocortex (*27*, *28*). These samples represent scRNA-seq experiments from different tissues, technical platforms, and developmental time scales. We further demonstrated the ability of *DeepVelo* to deconvolve gene coexpression networks on two additional data sources (mouse gastrulation, developing human forebrain) and benchmarked against linear and state-of-the-art vector-field learning approaches on out-of-sample velocity prediction accuracy (*16*, *26*, *29*).

## RESULTS

### A general framework using neural ODEs to model single-cell transcriptome dynamics

**Fig. 1.**
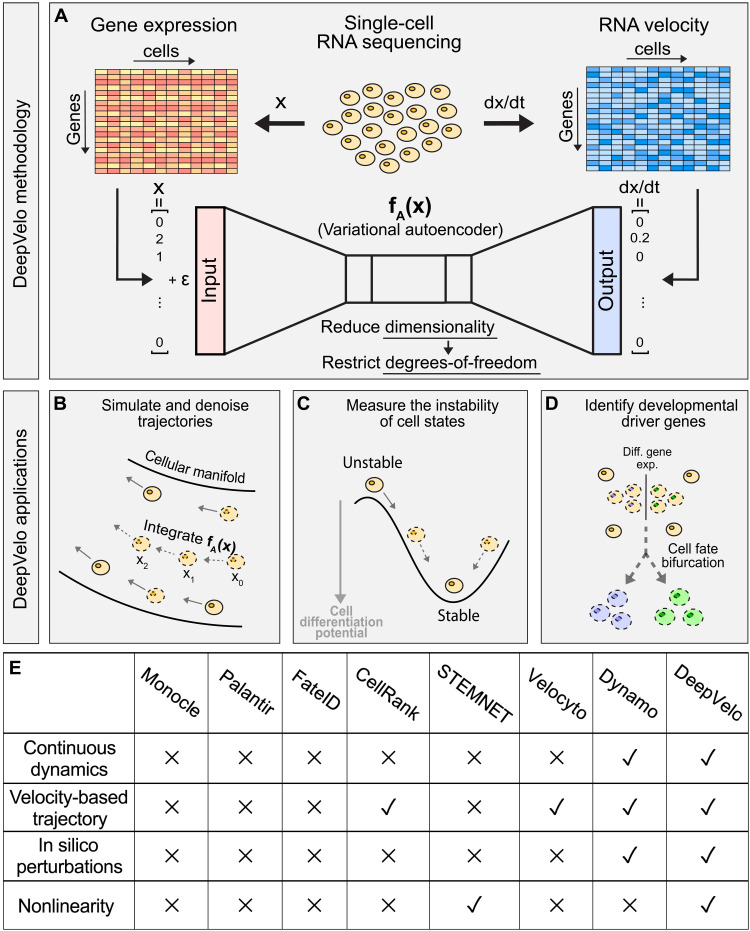
Schematic for *DeepVelo*. (**A**) Gene expression profiles and corresponding transcriptional velocities can be derived from scRNA-seq. After learning the mapping between gene expression and RNA velocity, the VAE represents a neural differential equation that encapsulates transcriptome dynamics. (**B**) Given an initial condition and time, our framework solves for the future gene expression state by integrating the VAE with a black-box ODE solver. (**C**) Our approach can simulate trajectories to evaluate the instability of cell states in a dynamical system. (**D**) *DeepVelo* can perform in silico perturbation studies to identify the developmental driver genes that determine the fate of cell bifurcations. (**E**) Comparison between *DeepVelo* and existing single-cell methods.

Our *DeepVelo* framework contains two main steps: inferring gene interaction and predicting continuous cell transitions. First, *DeepVelo* connects the gene expression profile of an individual cell ($\vec{x}$) to the instantaneous expression change with respect to time (RNA velocity $\frac{\partial \vec{x}}{\partial t}$). Distinct from existing methods that assume linear gene interactions (i.e., $\frac{\partial \vec{x}}{\partial t}=A\vec{x}$ with matrix ***A***), we train a VAE **f**_**A**_ to capture the nonlinear gene regulatory relationships (e.g., multiple TFs coactivating gene transcription) and map gene expression state to the RNA velocity, expressed by $\frac{\partial \vec{x}}{\partial t}=f_{A}(\vec{x})$ (Fig. 1, A and E). Consequently, the trained VAE **f**_**A**_ can directly estimate the instantaneous gene expression change of individual cells from any state (whether measured or not). Second, given some initial gene expression state close to the data, we can numerically compute the future (or past) gene expression states by integrating with any black-box ODE solver. For example, given gene expression state $\vec{x_{0}}$ at time *t* = 0, we can use Euler’s method, $\vec{x_{t+1}}=\vec{x_{t}}+f_{A}(\vec{x_{t}})$, to find the gene expression state at $\vec{x_{1}}$ and can iteratively perform this step for $\vec{x_{2}},\ldots ,\vec{x_{n}}$. As a result, *DeepVelo* can outline the developmental trajectory of single cells through time by sequentially computing the next gene expression state. Furthermore, with different initial conditions $\vec{x_{0}}$, our framework can derive detailed insights into the future (or past) of different cell states.

Here, we explored three major applications of *DeepVelo* in scRNA-seq data analysis. First, we simulated and denoised developmental trajectories by extrapolating the dynamics to out-of-sample cells (Fig. 1B). Second, we evaluated the instability of cell states by tracking gene expression changes along simulated trajectories (Fig. 1C). Third, we performed in silico studies to investigate how perturbations in initial gene expression conditions affect the fate of cell bifurcations (Fig. 1D).

### Predicting future cell states with learned neural ODEs

**Fig. 2.**
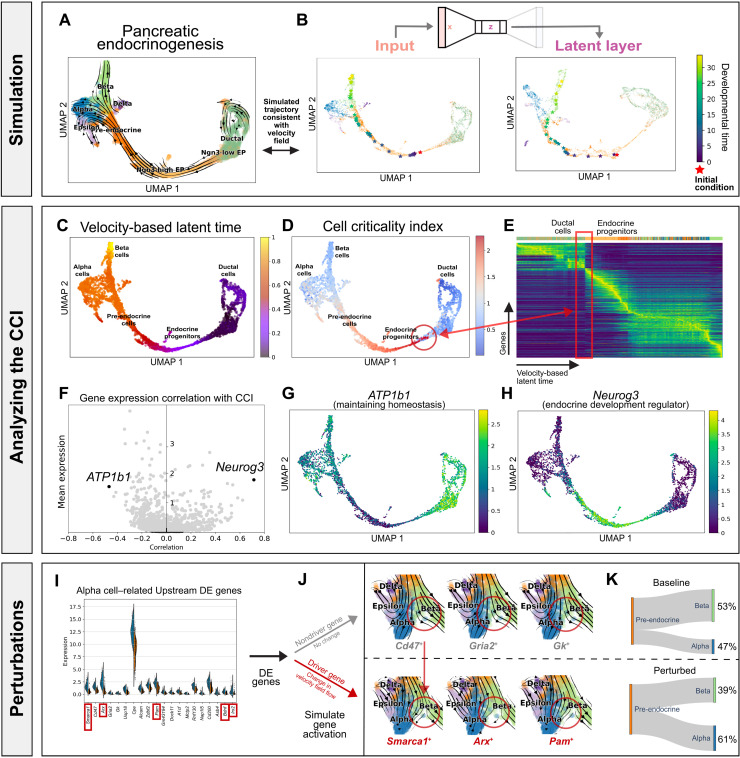
Pancreatic endocrinogenesis as a dynamical system. (**A**) Pancreatic endocrinogenesis phase portraits projected in a low-dimensional embedding. Here, each cell is represented by a gene expression state and an RNA velocity vector. (**B**) Simulating the developmental trajectory (in viridis) of an out-of-sample cell (in red) forward in time, visualized in a uniform manifold approximation and projection (UMAP) embedding of the gene expression state (*x*), autoencoder latent layer (*z*). (**C**) Velocity-based latent time derived from the RNA velocity for each cell. (**D**) The CCI derived from *DeepVelo* for each cell. (**E**) Gene expression of cells ordered by the velocity-derived latent time. Here, the CCI reveals unstable cell states indicative of fate commitment with a broad shift in gene expression patterns in latent time. (**F**) Genes that highly correlate with the CCI reveal driving forces behind endocrine progenitor dynamics. (**G**) In particular, *ATP1b1*, which negatively correlates with the CCI, has an important function in maintaining homeostasis in stable and stationary cell types. (**H**) *Neurog3*, which positively correlates with the CCI, is a known pre-endocrine master regulator. These genes support the CCI as a metric for the stability of single-cell states. (**I**) Differential gene expression analysis of pre-endocrine cells reveals key putative genes that correlate with the fate of transforming into an alpha versus a beta cell. (**J**) Visualizing the velocity field of perturbed cell states with activation in alpha cell–related driver genes versus nondriver genes. The red circles highlight differences in the velocity field. (**K**) Early perturbation of driver genes, which are a subset of DE genes, associated with an alpha cell fate resulted in a higher proportion of alpha cells from the pre-endocrine cells, suggesting a causal relationship through in silico studies.

To evaluate whether *DeepVelo* can uncover dynamics from sparse and noisy scRNA-seq experiments, we considered a mouse pancreatic endocrinogenesis dataset with transcriptomes profiled at embryonic day E15.5 from 10x Genomics (Fig. 2A) (*26*). Here, we show that summarizing the dynamics as neural ODEs can derive new insights about the data and further our understanding of pancreatic endocrinogenesis.

First, we examined a hypothetical trajectory simulated from *DeepVelo* after training the VAE on pancreatic endocrinogenesis cell states and velocities (Fig. 2B and fig. S1). When simulating hypothetical trajectories, future state predictions rely on out-of-sample cell states predicted from the previous time point. Hence, we evaluated the ability of *DeepVelo* to predict future cell states for an out-of-sample initial condition. We simulated an out-of-sample cell by adding noise to the gene expression state of an existing cell, thereby representing a cell state that did not previously exist in the data. With the out-of-sample cell as the initial condition (represented by the red star in Fig. 2B), *DeepVelo* can compute a series of future cell states along the developmental trajectory (represented by the stars transitioning from purple to yellow proportional to time in Fig. 2B). In pancreatic endocrinogenesis, the out-of-sample cell started as an endocrine progenitor, developed into a pre-endocrine cell, and ultimately became a beta cell. When we integrated the VAE with evenly distributed time increments, the distances between intermediate states reflect the magnitude of the RNA velocity vectors. Higher rates of change in gene expression generated more separated intermediate states. Conversely, lower rates of change produced a denser collection of intermediate points along the manifold.

During VAE training, regularized loss in the latent layer promotes a continuous, compact representation of locally similar cell states and velocities in the embedding (Fig. 2B). After training, the VAE latent embedding can be interpreted as a data manifold that combines information from gene expression states and velocities. Compared to gene expression embeddings, the latent embeddings can better disentangle distinct developmental trajectories because of the incorporation of velocity information. In pancreatic endocrinogenesis, the chronological and hierarchical orders of developmental trajectories are properly encoded in the latent layer (fig. S2). For example, ductal cells represent a major starting state, whereas alpha and beta cells represent major terminal states. These could be used to qualitatively assess the out-of-sample predictions made by *DeepVelo*. Because the output velocity is recursively added into the input cell states, it is also possible to see how future cell states emerge from previous predictions. For example, the simulated cell migrates along a trajectory spanned by the gene expression, latent, and velocity manifolds.

### Characterizing instability in *DeepVelo*-predicted trajectories with the cell criticality index

Next, we aimed to characterize the stable and unstable fixed points of this single-cell dynamical system. With only instantaneous velocity, *scVelo* could not track the gene expression changes of a single cell over a long period of time. In contrast, *DeepVelo* can simulate a continuously evolving hypothetical trajectory across time for each cell. By looking forward in time, we can numerically approximate the cell criticality index (CCI), which describes the future instability of single-cell states. For a cell, we define the CCI as the cumulative information change, or the cumulative Kullback-Leibler (KL) divergence, between gene expression distributions at each time step in the developmental trajectory. In other words, cell states that undergo large changes across time will have a high CCI, whereas cell states that go through only small changes will have a low CCI.

For each cell, we used *DeepVelo* to compute a developmental path such that the cell arrived at a steady terminal state. Then, we calculated the CCI along each path (Fig. 2D). The resulting developmental topology is similar to the classical Waddington landscape (*30*). In particular, the CCI can reveal unique topological information in the developmental landscape not directly observed in the velocity-based latent time estimated by *scVelo* (Fig. 2, C and E) (*31*). For example, the endocrine progenitor states exhibit a higher criticality, whereas the ductal cell and differentiated endocrine cell states experience a lower criticality. When ductal cells undergo transformation into islet cell types, the heightened criticality in endocrine progenitors represents fate commitment or a point of no return during development. In dynamical systems, this behavior suggests that cell states with low criticality are located at a stable fixed point, with the cell identity remaining stable against small gene expression perturbations. More interestingly, the endocrine progenitors are located at an unstable fixed point with properties similar to those of a chaotic system in which a small perturbation may result in large downstream changes. We can substantiate the instability of cell states by examining the genes that best correlate with the CCI (Fig. 2F). For example, previous experiments have shown that *Neurog3*, which positively correlates with the CCI, is a known driver for endocrine commitment (Fig. 2H), and *ATP1b1*, which negatively correlates with the CCI, is important for maintaining homeostasis in stable and stationary cell types (Fig. 2G) (*32*, *33*). The expression of these genes supports the CCI as a metric for evaluating the instability of single-cell states. Furthermore, when viewed in conjunction with velocity-based latent time, the CCI can provide new insights into the relative timing of critical cell states in a developmental process.

### Conducting in silico perturbation studies with *DeepVelo*

Last, we investigated the behavior of this dynamical system with perturbation studies (*34*). The goal of in silico perturbation studies is to computationally identify which initial gene expression conditions affect the fate of cell bifurcations. In short, we used pre-endocrine cells (*n* = 592) as the initial conditions and trained a *K*-nearest neighbors (KNN) classifier (*K* = 30) to identify the postbifurcation cell type. By allowing these pre-endocrine cells to naturally evolve according to the dynamics learned by *DeepVelo*, we observed a baseline 1:1.57 ratio of terminal alpha versus beta cell states, similar to the proportion of alpha and beta cells in the mouse pancreas (*35*). The ratio of terminal cell states indicates that the beta cell state is a stronger attractive terminal state than the alpha cell state, which corroborates with previous conclusions (*31*). Then, we examined upstream differential expression (DE) between initial pre-endocrine cells of different fates (Fig. 2I). The results suggest that early expression perturbations in key upstream genes correlate with the fate of developmental bifurcations.

Further, we formulated a way to perform hypothesis testing and to infer causal relationships at developmental branching points (*36*). We hypothesize that fate-determining driver genes are a subset of upstream DE genes (Fig. 2J). To investigate which developmental driver genes can independently cause progenitor cells to prefer one trajectory over another, we strategically activated the expression of one alpha cell–related DE gene at a time in pre-endocrine cells, similar to the perturbation experiments in *dynamo* (*21*). Specifically, we postulate that perturbations in driver genes could lead to a larger proportion of alpha cells as terminal cell states. We observed that 5 of the top 20 DE genes resulted in a statistically significant increase (binomial test *P* < 0.05) in the proportion of alpha cells compared with the baseline with the dynamics learned by *DeepVelo* (Fig. 2K and table S1). Overall, the average ratio of alpha versus beta cells increased to 1.69:1. Thus, in silico perturbation studies can be used to efficiently and comprehensively identify developmental driver genes upstream of the signaling cascade. More interestingly, simulation results suggest that pancreatic endocrinogenesis development exhibits properties similar to chaotic systems, where small perturbations in key driver genes determine the fate of cell bifurcations. In other words, small variations in the initial conditions of a cell may result in large downstream changes.

### Exploring the neural ODEs governing the developing mouse dentate gyrus

**Fig. 3.**
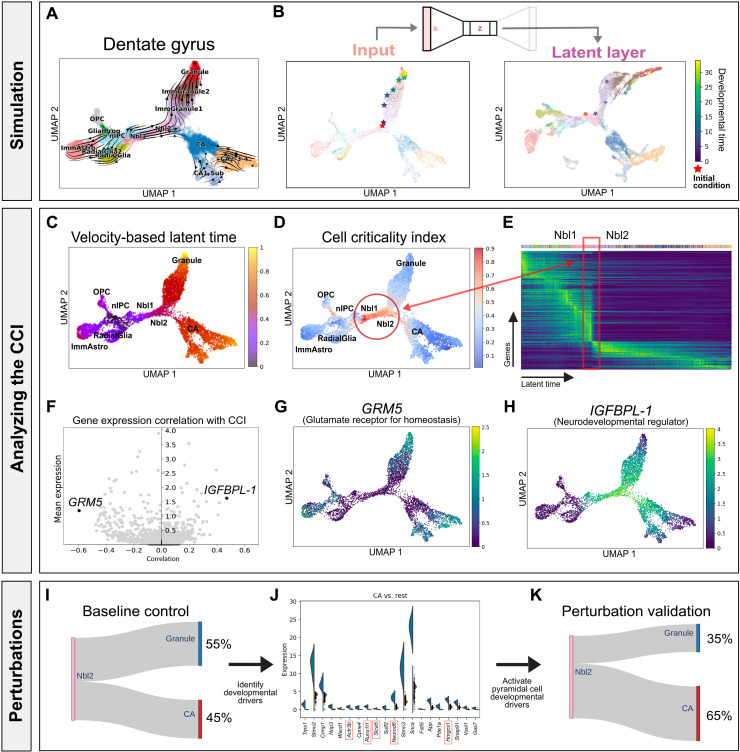
Developing mouse dentate gyrus as a dynamical system. (**A**) Dentate gyrus phase portraits projected in a low-dimensional embedding. Each cell is represented by a gene expression state vector and an RNA velocity vector. (**B**) Simulating the developmental trajectory (in viridis) of an out-of-sample cell (in red) forward in developmental time, visualized in UMAP embeddings. (**C**) Velocity-based latent time derived from *scVelo* for each cell. (**D**) CCI derived from *DeepVelo* for each cell. (**E**) The CCI reveals instability during the transition from *Nbl1* to *Nbl2* cells, indicative of cell fate commitment. (**F**) Genes that highly correlate with the CCI reveal driving forces behind the dentate gyrus dynamics. (**G**) In particular, *GRM5*, which shows the strongest negative correlation with the CCI, encodes glutamate receptors in stable and stationary neurons. (**H**) *IGFBPL-1*, which shows the strongest positive correlation with the CCI, regulates neurodevelopment. (**I**) Baseline proportion of terminal cell types that result from *Nbl2* cells. (**J**) Differential gene expression analysis of *Nbl2* cells reveals key putative genes that correlate with the fate of transforming into a pyramidal versus granule cell. Early perturbation of each top DE genes associated with a pyramidal cell fate can result in a statistically significant (highlighted in red) higher proportion of pyramidal cells, suggesting a causal relationship through in silico studies. (**K**) Average terminal cell ratios after in silico perturbations.

Here, we further evaluated whether *DeepVelo* can uncover the dynamics of a scRNA-seq dataset from a different tissue, developmental time scale, and technical platform. Specifically, we considered an scRNA-seq dataset of the developing mouse dentate gyrus with transcriptomes profiled using droplet-based scRNA-seq (Fig. 3A) (*27*). After obtaining a neural network representation of the dentate gyrus dynamics, we simulated an out-of-sample cell by perturbing the gene expression state of an *Nbl2* cell. With the out-of-sample cell as the initial condition, we used *DeepVelo* to simulate a trajectory into the future, which moved along the existing granule cell trajectory in the data (Fig. 3B). Furthermore, the VAE embeddings properly encoded the developmental hierarchy of cell types in the low-dimensional dynamic manifold.

When examining critical cell states in the dentate gyrus, we observed an abrupt gene expression shift in the developmental manifold, which can be visualized by ordering cells in latent time derived from *scVelo* (Fig. 3C). Specifically, the change in gene expression marks the transition from *Nbl1* to *Nbl2* cells and suggests fate commitment during the transition (Fig. 3D). After calculating the CCI, we found that cells experiencing this abrupt change also have a high criticality, which substantiates the CCI as a metric for quantifying the instability of cell states (Fig. 3E). In addition, the most strongly correlated genes in the dentate gyrus highlight the robustness of the CCI as an instability measure (Fig. 3F). For example, *IGFBPL-1* shows the strongest positive correlation with the CCI, and it has been reported to regulate neuron differentiation in progenitor cells (Fig. 3H). *GRM5* is the most negatively correlated gene with CCI. Consequently, *GRM5* also encodes glutamate receptors in stable and differentiated neurons (Fig. 3G) (*37*, *38*).

Last, we conducted in silico perturbation studies to determine the genetic drivers governing dentate gyrus cell fate decisions. We allowed the upstream *Nbl2* cell states (*n* = 1003) to naturally evolve according to the dynamics captured by *DeepVelo*, which resulted in either terminal granule or pyramidal cell states categorized by a KNN classifier (Fig. 3I). The ratio of simulated granule versus pyramidal terminal cell states was 1.2:1, similar to the ratio of cell states measured in the assay. Then, we performed DE analysis on the initial conditions (i.e., the *Nbl2* cell states) of different fates (Fig. 3J). *DeepVelo* identified the pyramidal neuron developmental driver gene *Runx1t1*, which was recently shown to induce pyramidal neuron formation, with its deletion resulting in reduced neuron differentiation in vitro (*39*). In addition, *Prox1* is a DE gene associated with a granule cell fate. This gene was previously identified by *velocyto* and experimentally validated as being necessary for granule cell formation; moreover, the deletion of *Prox1* leads to adoption of the pyramidal neuron fate (*37*). As further validation and to identify developmental driver genes, we increased the expression of each pyramidal neuron–related upstream DE gene in *Nbl2* cells and observed an elevated proportion of pyramidal neurons as terminal cells under the dynamics captured by *DeepVelo*. Overall, 5 of the top 20 genes resulted in a statistically significant increase (binomial test *P* < 0.05) in the proportion of pyramidal cells (Fig. 3K and table S2). In summary, in silico perturbation studies can provide a low-cost alternative for identifying developmental driver genes using datasets from different tissues, developmental time scales, and technical platforms.

### Formulating neural ODEs underlying the mouse neocortex across multiple embryonic days

**Fig. 4.**
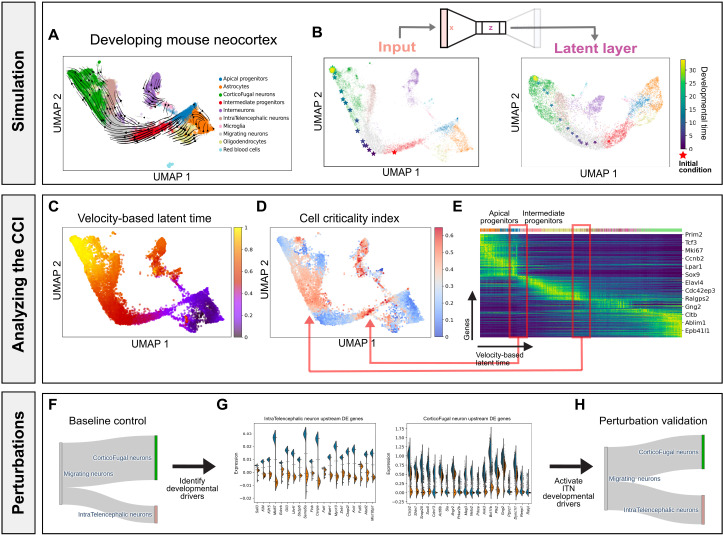
Developing mouse neocortex over multiple embryonic days. (**A**) Mouse neocortex phase portraits projected in a low-dimensional embedding. (**B**) Simulating the trajectory (in viridis) of an out-of-sample cell (in red) forward in developmental time, visualized in gene expression embeddings and VAE latent layer embeddings. (**C**) Velocity-based latent time derived from *scVelo*. (**D**) The CCI reveals two unstable fixed points indicative of a biphasic fate-commitment dynamic. (**E**) One latent time point highlights the transition from APs to IPs, and the other highlights the transition from MNs to CFNs. (**F**) Baseline proportion of terminal cell types from MNs. (**G**) Differential gene expression analysis of the simulated MNs reveals key putative genes that correlate with the fate of transforming into an ITN versus CFN. (**H**) Early perturbation of the top DE genes associated with a pyramidal cell fate resulted in an average 10% higher proportion of ITNs from the perturbed MNs.

We evaluated whether *DeepVelo* can learn the dynamics underlying a more complex developmental process. We examined an scRNA-seq experiment of the developing mouse neocortex with transcriptomes profiled at embryonic days E14, E15, E16, and E17 (Fig. 4A) (*28*). After batch correction and velocity-field learning (fig. S3), we generated an out-of-sample intermediate progenitor (IP) as the initial condition (Fig. 4B). The simulated developmental trajectory suggests that the hypothetical IP transitioned into a migrating neuron (MN) and then subsequently became a CorticoFugal neuron (CFN). The distance between each time step was larger when the simulated cell was an IP compared with an MN, indicating that the IPs transition at a faster rate than the MNs. The latent layer embeddings also properly encoded the progression from neural progenitors to differentiated neurons (Fig. 4B).

When analyzed in conjunction with velocity-based latent time, we observed a biphasic fate-commitment dynamic with high criticality among IPs and MNs but low criticality in apical progenitors (APs) through the CCI (Fig. 4, C to E). This finding suggests that APs play a more important role in self-renewal, whereas the IPs are preprogrammed for neuronal differentiation through a broader shift in gene expression patterns. In addition, as MNs branch into IntraTelencephalic Neurons (ITNs) and CFNs, *DeepVelo* can effectively disentangle the underlying bifurcation dynamics. When we allowed a set of MNs (*n* = 1000) to evolve according to the dynamics learned by *DeepVelo*, we observed a baseline 3:7 ratio of ITNs versus CFNs (Fig. 4F). After perturbing the initial DE genes associated with an ITN fate on the same set of MNs (Fig. 4G), we observed an average 10% increase in ITN proportion compared with the baseline (Fig. 4H). These observations indicate that *DeepVelo* can accurately learn an overarching neural ODE of mouse neocortex gene expression dynamics spanning several embryonic days. Furthermore, these results suggest that our framework can identify driver genes that play crucial roles in developmental dynamics with a continuous representation of velocity fields.

### Reconstructing gene coexpression networks with retrograde trajectories

Gene coexpression networks have been widely used to connect genes of unknown function to biological processes, discern transcriptional regulatory relationships, and prioritize candidate genes for genetic disorders. scRNA-seq experiments are particularly useful in dissecting these networks because of the single-cell resolution and cell type specificity. However, it is computationally challenging to detect strong correlations between genes using scRNA-seq data due to the noisy and sparse transcript measurements in single-cell experiments (*40*–*42*).

**Fig. 5.**
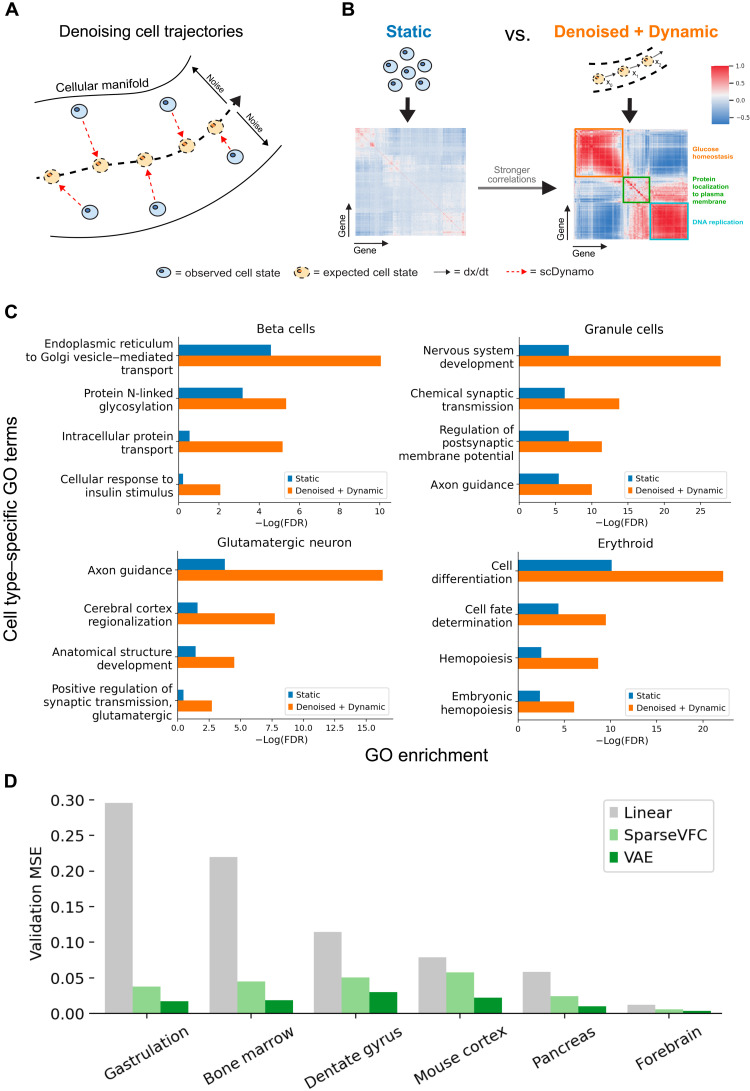
Disentangling trajectory-specific gene coexpression networks. (**A**) Schematic for reducing variability along a developmental trajectory due to sparsity and noise in scRNA-seq experiments with denoising VAEs in *DeepVelo*. (**B**) We used a representative initial condition (e.g., the median expression profile for a cluster of cells) to simulate denoised cell trajectories according to the dynamics learned by *DeepVelo*. Compared with static cell clusters, the dynamic cells in denoised trajectories have stronger correlations between genes, which leads to better coherence between functional gene modules. Given the same set of genes, the two correlation matrices are each biclustered to identify functional gene coexpression modules. (**C**) GO enrichment of cell type–specific terms from the most significant gene cluster. Gene coexpression modules from dynamic cells have higher cell type–specific GO term enrichment than gene modules found from existing methods. (**D**) Benchmarking with the linear and state-of-the-art vector-field learning method *SparseVFC* shows that our VAE-based framework can improve out-of-sample velocity vector prediction accuracy by at least 50% across the six datasets, indicating that *DeepVelo* can learn a more accurate representation of the velocity field.

Therefore, we propose to use *DeepVelo*’s denoising VAE to reduce the variability along a developmental trajectory caused by the sparsity and noise in scRNA-seq data (Fig. 5A and fig. S4). To account for the tissue specificity, we used differentiated (or terminal) beta cells as the initial conditions and reversed the developmental time using *DeepVelo*. As a result, the retrograde developmental trajectory represents the gene dynamics that would have resulted in the terminal cell types. To compare the robustness of gene coexpression networks from *DeepVelo* versus scRNA-seq, we performed gene ontology (GO) analysis after biclustering the coexpression matrices with the same genes. We found that the gene coexpression modules from retrograde trajectories have more significant correlations compared to the gene modules from static cells. Furthermore, we postulate that more coherence within gene modules would form clusters with higher enrichment on cell type–specific GO terms (Fig. 5B). Benchmarking on four datasets shows that functional gene modules found from denoised and dynamic cells in retrograde trajectories have at least two orders of magnitude higher enrichment for cell type–specific GO terms than static cell clusters (Fig. 5C). Therefore, the retrograde trajectories computed by *DeepVelo* effectively disentangle trajectory-specific gene regulatory networks and can provide a computational solution for boosting signal-to-noise ratios in single-cell gene coexpression networks.

### Comparing *DeepVelo* with existing methods

*DeepVelo* qualitatively differs from existing ODE-based regulatory networks (*43*). First, explicitly deriving differential equations for biological processes is only feasible when examining small-scale systems (*44*–*47*). In contrast, *DeepVelo* can capture high-dimensional interactions and can scale to a large number of variables. Second, *DeepVelo* uses a neural network to learn potentially nonlinear gene interactions, which is more suitable for modeling complex biological processes compared with linear ODEs and other kernel-based sparse approximation methods (*48*, *49*). In particular, we compared *DeepVelo* with the linear method and the state-of-the-art vector-field learning approach *SparseVFC* (*21*). Given the same training data with gene expression state as input and RNA velocity as the ground truth output, we evaluated the accuracy of the RNA velocity predictions using mean square errors. Benchmarking results on six datasets show that our method can reduce the RNA velocity prediction error by at least 50%, indicating that *DeepVelo* can provide a more accurate representation of velocity vector fields and compute future cell states with better numerical precision (Fig. 5D and fig. S5). In terms of runtime and peak memory usage, *DeepVelo* can also be efficiently accelerated with graphic-processing units (fig. S6). Last, many previous ODE-based methods use pseudotime as a substitute for time. In comparison, *DeepVelo* uses RNA velocity, which reflects unit time on the scale of hours (*20*).

## DISCUSSION

Recent advances in single-cell sequencing technologies have opened new avenues for investigating a fundamental question in biology: How do individual cells undergo precise and highly coordinated regulation to become complex tissues? Previously, computationally modeling these continuous biological processes was challenging because of the sparse, noisy RNA measurements, complex gene regulatory relationships, and destructive nature of many sequencing experiments (e.g., can only capture a snapshot of the developmental trajectory). Here, we propose *DeepVelo*, a computational framework that can learn to model the underlying nonlinear, continuous-time gene expression dynamics of transitioning single cells.

Distinct from existing tools that view single-cell datasets as a static manifold (*13*, *14*, *50*), *DeepVelo* treat individual cells as a dynamic system continuously transitioning from one state to another. Hence, by deriving accurate differential equations that quantify the gene expression dynamics of single cells, *DeepVelo* can answer many questions about cell fates and the genetic drivers governing developmental trajectories. In addition, numerous studies have reported that gene regulatory relationships are highly tissue-specific and nonlinear. For instance, multiple TFs can both compete and collaborate to use several proximal and distal cis-regulatory elements to initialize gene transcription (*51*, *52*). Existing methods often ignore these nonlinear relationships and thus may fail to accurately predict expression changes from existing RNA measurements. In contrast, *DeepVelo* leverages a deep neural network to capture complex gene interactions in a data-driven manner, allowing more precise expression-to-velocity connections. Currently, *DeepVelo* only models gene expression dynamics within a single cell. A future direction could expand the state space of *DeepVelo* and incorporate gene interactions between spatially neighboring cells using spatial transcriptomics (*53*).

We demonstrated the benefit of our *DeepVelo* model via three applications. First, we showed *DeepVelo*’s ability to simulate accurate cell trajectories on out-of-sample cells. Furthermore, by reversing the developmental time of differentiated cells, retrograde trajectories can deconvolute trajectory-specific gene coexpression networks and discover more coherent cell type–specific gene modules. Second, we designed a generalizable metric called the CCI, analogous to the “kinetic energy” of a Waddington landscape, to accurately measure the magnitude of gene expression changes along developmental paths (*30*). In our analysis, we demonstrated that the CCI could highlight critical fixed points in single-cell dynamical systems. It is worth noting that instantaneous higher-order derivatives of velocity computed from *dynamo* are different from the integral-based CCI that captures changes across long periods of time. Third, we performed in silico gene perturbation studies in the context of developmental dynamical systems, which is different from existing tools that predict experimental perturbations such as *scGEN* and *MELD* (*54*, *55*).

In summary, we introduce the computational framework *DeepVelo* to model continuous-time transcriptional dynamics from scRNA-seq data. *DeepVelo* has demonstrated potential for dissecting regulatory trajectories, highlighting critical cell states, and prioritizing developmental driver genes. Potential directions for future improvement include incorporating other modalities for regulation relationship inference and formulating the model as neural stochastic differential equations. With the exponential growth of scRNA-seq data, *DeepVelo* can be a useful tool for the community to illuminate the rules governing cell state dynamics and cell fates.

## MATERIALS AND METHODS

### Data collection and preprocessing

scRNA-seq data (mouse pancreatic endocrinogenesis, dentate gyrus, gastrulation, neocortex, and human forebrain) were downloaded from the National Center for Biotechnology Information (NCBI) Gene Expression Omnibus repository and the Sequence Read Archive. The raw FASTQ files were preprocessed using *Cellranger* v6.0.1 with default parameters (*56*). As references, we used GRCh38 (2020-A) for human samples and mm10 (2020-A) for mouse samples. After preprocessing, we obtained an mRNA count matrix with rows as cells and columns as genes. Then, using the *Cellranger* intermediate outputs, we computed the amount of spliced and unspliced mRNA transcripts using the *velocyto* package with default parameters (*16*). The output was a loom file with spliced and unspliced mRNA counts for each cell and gene.

For preprocessing and computing RNA velocities, we followed the procedure recommended by *scVelo* (*31*). We selected the top 3000 highly variable genes with at least 20 mRNA reads and normalized the mRNA counts within each cell (using the *scv*. *pp*. *filter*_*and*_*normalize* function in *scVelo*). The normalized mRNA count matrix became the gene expression state matrix. Then, we log-transformed the normalized spliced and unspliced counts for moment calculations. First- and second-order moments were computed using the top 30 principal components (PCs) and the top 30 nearest neighbors (using the *scv*. *pp*. *moments* function in *scVelo*). After recovering the dynamics using the moments, RNA velocity was computed with the generalized dynamical model from the raw normalized reads (using the *scv*. *tl*. *velocity* function and *mode* = ^”^*dynamical*^”^ setting in *scVelo*). Only the velocity genes were used as features for the neural ODE. The numbers of cells and available velocity genes for each dataset are shown in Table 1.

**Table 1.** Summary of scRNA-seq datasets used in this study.

**Organism**	**Tissue**	**Number of cells**	**# of velocity genes**
Mouse	Pancreatic endocrinogenesis	3,696	1,027
Mouse	Dentate gyrus	18,213	1,304
Mouse	Gastrulation	89,267	592
Mouse	Neocortex	32,061	2,444
Human	Forebrain	1,720	826

### VAE architecture

High-dimensional single-cell dynamical systems are difficult to model due to high degrees of freedom. For example, the number of features can sometimes be larger than the number of data points. Consequently, gene expression would only vary in a small portion of dimensions. Therefore, modeling the gene expression dynamics of a low-dimensional manifold embedded in high-dimensional data is a challenging task. Consequently, autoencoders can reduce the dimensionality of the data by introducing an information bottleneck. Accordingly, when used to represent dynamical systems, autoencoders can restrict cell transitions to only movements along the low-dimensional manifold.

A VAE consists of an encoder, which parametrizes Gaussian distributions to be sampled from, and a decoder, which transforms the sampled values into the output. For the encoder and the decoder, four dense layers (size 64 as the intermediate layer and size 16 as the latent layer) with *relu* activation were constructed using the Tensorflow and Keras packages (*57*, *58*). The VAE takes the gene expression state as input, and outputs the RNA velocity. In the VAE, the encoder layers with weights *W_e_* and biases *b_e_* produce the hidden layer *h*(*x*), which parametrizes the location and scale of *i* Gaussian distributions. Then, a sample from each reparametrized Gaussian distribution *z_i_* is used as input for the decoder layer with weights *W_d_* and biases *b_d_*. The architecture can be expressed as

EncoderLayer(x)=h(x) (1)

$$=\text{Relu}(b_{e}+W_{e}*x)$$ (2)

$$\mu_{i}(x)=\text{EncoderLayer}(h(x))$$ (3)

$${\sigma^{2}}_{i}(x)=\text{EncoderLayer}(h(x))$$ (4)

$$\epsilon_{i}\sim N(0,I)$$ (5)

$$\;z_{i}\sim \mu_{i}(\frac{\partial x}{\partial t})+\epsilon_{i}*{\sigma^{2}}_{i}\;(\frac{\partial x}{\partial t})$$ (6)

$$\text{DecoderLayer}(z_{i})=\text{Relu}(b_{d}+W_{d}*z_{i})$$ (7)

where the *Relu*(z) activation function is

Relu(z)=max(0,z) (8)

We used the mean squared error as the reconstruction loss and minimized the loss with the Adam optimizer. To prevent overfitting and to encourage a sparse representation of latent embeddings, L1 regularization was added to the activation of all layers with λ = 1 × 10^−6^. The evidence lower bound loss function with $\left(z|\frac{\partial x}{\partial t})=N(\mu_{i}(\frac{\partial x}{\partial t}),\text{diag}({\sigma^{2}}_{i}(\frac{\partial x}{\partial t}))\right)$ *p*(*z*) = *N*(0, *I*), can be described as

$$L(\frac{\partial x}{\partial t},\hat{\frac{\partial x}{\partial t}})=\text{KL}\;\text{Divergence}+\text{Reconstruction Loss}$$ (9)

$$=\mathrm{KL}(q(z|\frac{\partial x}{\partial t})\;\|\;p(z))-\sum_{i=1}^{D}{\left(\frac{\partial x}{\partial t}-\hat{\frac{\partial x}{\partial t}}\right)}^{2}$$ (10)

Because the input and output vectors are sparse, a small learning rate of 0.00001 was used with a batch size of 32. Early stopping was added once the validation loss did not improve for three consecutive epochs (fig. S7).

### Initial value problems and ODE solvers for integration

Our framework can be used to predict gene expression profiles across time. Given *t*_0_ and $\vec{x}(0)=x_{0}$, this is an initial value problem with the goal of solving $\vec{x}(t)=\vec{x_{t}}$ for any *t*

$$\frac{\partial \vec{x}(t)}{\partial t}=f(t,\vec{x}(t))$$ (11)

Here, f is only a function of the state $\vec{x_{t}}$ such that $f=f_{A}(\vec{x_{t}})$. Then, the equation becomes

$$\frac{\partial \vec{x_{t}}}{\partial t}=f_{A}(\vec{x_{t}})$$ (12)

The first-order Euler’s method for finding the state $\vec{x_{t+1}}$ is

$$\vec{x_{t+1}}=\vec{x_{t}}+\frac{\partial \vec{x_{t}}}{\partial t}$$ (13)

$$=\vec{x_{t}}+f_{A}(\vec{x_{t}})$$ (14)

However, we can use higher-order ODE solvers from the SciPy package to find a more accurate solution (*59*). The explicit Runge-Kutta method of order 8 (DOP853) was used to obtain the most accurate solutions, but it has a slow runtime. The explicit Runge-Kutta method of order 3 (RK23) can be used to trade off accuracy for a faster runtime.

From the preprocessing steps, gene expression state *x* is the normalized mRNA count, and RNA velocity $\frac{\partial x}{\partial t}$ is the rate of change of the normalized mRNA counts. The predicted RNA velocity should be linearly scaled before being used to approximate numerical derivatives with respect to time for integration. We estimated that a velocity scaling factor of 0.65 ± 0.05 worked well in terms of our normalization and integration procedures. Furthermore, to estimate the maximum number of steps to integrate to reach the terminal state of a cell, we computed the 2 SD range for the expression of each gene. Then, we divided the range of each gene by the mean velocity to find the step size of each gene. The maximum step size is defined as the 95th to 99th percentile of the step sizes. More simply, the maximum step size would allow a 2 SD change in the expression of at least 95% of the genes.

### Addressing drift effects

In control theory, using only the previous state and the velocity vectors to predict the next state can result in a phenomenon called “dead reckoning,” where the errors accumulate after each step (*60*). Under our framework, RNA velocity prediction errors could come from many factors. For example, the VAE may not have enough internal (e.g., genes) or external (e.g., environmental) features to accurately predict RNA velocities. The black-box integration procedures may also introduce numerical errors. To mitigate this effect, we used two strategies

1) Instead of a traditional VAE, we trained a denoising VAE to reduce the variance of the predicted RNA velocity. By adding a small exponentially distributed noise ϵ (λ < 10^4^) to the gene expression read count inputs during training, we could increase the generalizability of the input space and improve extrapolations to out-of-sample cell states.

$$\frac{\partial \vec{x}}{\partial t}=f_{A}(\vec{x}+\epsilon )$$ (15)

2) As we integrated the VAE over some time, we found reference cells in the data manifold every few steps and continued integration from the reference cell, as a form of a high-gain Kalman filter. We designated the intermediate step size as a hyperparameter relative to the step size. For example, after integrating for five intermediate steps, we projected the predicted (or extrapolated) gene expression state to the original dataset using the top 30 PCs. Then, we identified the KNN (*K* = 5) within the principal components analysis (PCA) embeddings. The reference cell is defined as the mean expression profile among those KNN cells from the dataset, and ODE integration continued from this reference cell. This allowed our prediction to adhere closely to the data manifold and further reduced the degree of freedom. Consequently, finding reference cells in the data also constructed boundary conditions when integrating a dynamical system. For example, once the extrapolated state went beyond the cellular manifold, there were no cells in the data to serve as a reference, but the nearest neighboring cells from the dataset could still construct a reference cell from where integration could continue.

### Classifying future cell states simulated by *DeepVelo*

Given an initial cell state, we can integrate the autoencoder to find future cell states. To identify the cell types of the cell states simulated by *DeepVelo*, we trained a nonparametric KNN classifier (*K* = 30) that takes the top 30 PCs of the gene expression states as input and outputs the cell type labels (*61*). For each new cell state, the gene expression vector is projected onto the same top 30 PCs, which is passed to the trained KNN classifier for cell type prediction. The output is a zero to one probability of whether the cell belongs to each cell type. The new cell state is categorized under the cell type with the highest predicted probability.

### Measuring instability with the CCI

By solving for the developmental path of a single cell, we can measure the amount of gene expression change along a trajectory, rather than comparing only the difference between the start and end states. Previously, StemID used the entropy of the gene expression distribution to heuristically identify stem cells in single-cell transcriptome data, where pluripotent cells tend to have a more uniform gene expression distribution with a higher entropy and differentiated cells tend to have a lower entropy (*62*). If $\vec{x^{g}}$ denotes the expression state of the genes *g*, then the StemID of the gene expression state is defined as

$$\text{StemID}(\vec{x})=-\sum_{i\in g}x^{i}\text{log}(x^{i})$$ (16)

We reasoned that a change in the gene expression distribution (e.g., from high to low entropy) can be captured using the relative entropy (or the KL divergence). On the basis of this idea, we devised a measure to quantify the capacity for any cell to undergo a gene expression change in the dynamical system. The CCI is calculated as the cumulative information change or the cumulative KL divergence between gene expression distributions at each step in the developmental trajectory. Different from StemID, the CCI can quantify the gene expression change of a cell regardless of the pluripotency (fig. S8). As an analogy, StemID measures the “potential energy” of a cell’s ability to differentiate, whereas the CCI measures the kinetic energy of a cell’s ability to change. If $\vec{x_{t}^{g}}$ denotes the expression state of the genes *g* at time *t*, then the cumulative KL divergence for *T* = 35 steps can be defined as

$$\mathrm{CCI}(\vec{x})=\sum_{t=0}^{T}\mathrm{KL}(\vec{x_{t+1}^{g}}\;\|\;\vec{x_{t}^{g}})$$ (17)

$$=\sum_{t=0}^{T}\sum_{i\in g}x_{t+1}^{i}\text{log}(\frac{x_{t+1}^{i}}{x_{t}^{i}})$$ (18)

### Sampling out-of-sample cells and simulating perturbations

To sample initial gene expression states, we computed the median expression profile of a certain cell type (e.g., pre-endocrine progenitors in pancreatic endocrinogenesis) and added exponentially distributed noise using the variance of each gene within that cell type to randomly increase or decrease gene expression. To simulate perturbation in one gene, the 99th percentile expression level of that gene was applied to the initial conditions and subsequently to all the intermediate states. Terminal cell identity was determined by projecting the data onto the top 30 PCs and classified by using a KNN classifier (with *K* = 30). With the *scVelo* package, the dynamical mode estimates a variance for each gene over all cells, whereas the stochastic mode estimates a variance for each cell. Note that to model stochasticity in the stochastic mode, our framework could be easily adapted to also learn the variance of the velocity vectors (as neural stochastic ODEs).

### In silico perturbation studies

We divided the in silico perturbation study into three steps:

1) The upstream progenitor cell gene expression states were selected as initial conditions. First, we solved all of the initial gene expression states over time to establish a developmental baseline. Specifically, we aimed to observe the natural proportion of terminal cell types that could arise from the dynamical system without any intervention.

2) Then, we identified DE genes in the initial gene expression states that correlate with development into a particular terminal cell type later in time. Differential gene expression was performed using the *scanpy* package with the Wilcoxon test and Bonferroni corrections (*63*).

3) Last, we perturbed one trajectory-specific DE gene at a time in the initial gene expression states. The subset of upstream DE genes that resulted in a statistically significant increase (via a binomial test) in the proportion of cells toward the terminal cell type are categorized as developmental driver genes.

### Retrograde trajectory simulation

Similar to the in silico perturbation studies, we computed the median expression profile of a terminal cell type (e.g., beta cells, granule cells, glutamatergic neurons, or erythroid cells) in each scRNA-seq experiment (mouse pancreatic endocrinogenesis, dentate gyrus, human forebrain, and mouse gastrulation) as the representative initial condition. A set of cells (*n* = 50) was sampled from each representative initial condition by adding exponentially distributed noise using the variance of gene expression of the terminal cell type. The retrograde trajectory for each cell was simulated by subtracting the predicted RNA velocities from the gene expression state during integration

$$\vec{x_{t-1}}=\vec{x_{t}}-\frac{\partial \vec{x_{t}}}{\partial t}$$ (19)

$$=\vec{x_{t}}-f_{A}(\vec{x_{t}})$$ (20)

After integrating for 15 discrete steps each with 5 intermediate steps, a gene correlation matrix of the cells in retrograde trajectories was calculated.

### GO enrichment analysis

Hierarchical biclustering was performed on the coexpression matrices, and three gene clusters were identified from each coexpression matrix, representing three functional modules. We performed GO enrichment analysis on each functional module using GOATOOLS (*64*). To construct the background, we used the NCBI protein-coding genes (www.ncbi.nlm.nih.gov/gene/) for human (Taxonomy ID 9606) or mouse (Taxonomy ID 10090). We intersected the NCBI protein-coding genes with genes expressed by at least 10 cells in each scRNA-seq dataset to create a tissue-specific background. In addition, we used the GOs from http://geneontology.org/ontology/go-basic.obo and gene associations (file named gene2go.gz) from https://ftp.ncbi.nlm.nih.gov/gene/DATA/. After performing Fisher’s exact test, we calculated the Benjamini-Hochberg false discovery rates to correct for multiple testing. To compare between two coexpression matrices, we considered the most significant enrichment out of the three clusters for each GO term. In Figs. 3A and 5A, the most significantly enriched GO terms associated with biological processes are listed next to each gene cluster.

## Supplementary Materials

### This PDF file includes:

Figs. S1 to S8 Tables S1 and S2 References

## Notes

View/request a protocol for this paper from *Bio-protocol*.
