# Supplementary material for: DeepVelo: Single-cell transcriptomic deep velocity field learning with neural ordinary differential equations
Source: Sci Adv. 2022 Nov 30;8(48):eabq3745. doi: 10.1126/sciadv.abq3745 (PMC9710871; doi:10.1126/sciadv.abq3745)
Supplement: Supplementary file 1 — Figs. S1 to S8 Tables S1 and S2 References [file sciadv.abq3745_sm.pdf]

Science Advances
AAAS

Supplementary Materials for

DeepVelo: Single-cell transcriptomic deep velocity field learning with neural ordinary differential equations

Zhanlin Chen *et al.*

Corresponding author: Mark Gerstein, mark@gersteinlab.org; Jing Zhang, zhang.jing@uci.edu

Sci. Adv. **8**, eabq3745 (2022)
DOI: 10.1126/sciadv.abq3745

This PDF file includes:

Figs. S1 to S8
Tables S1 and S2
References

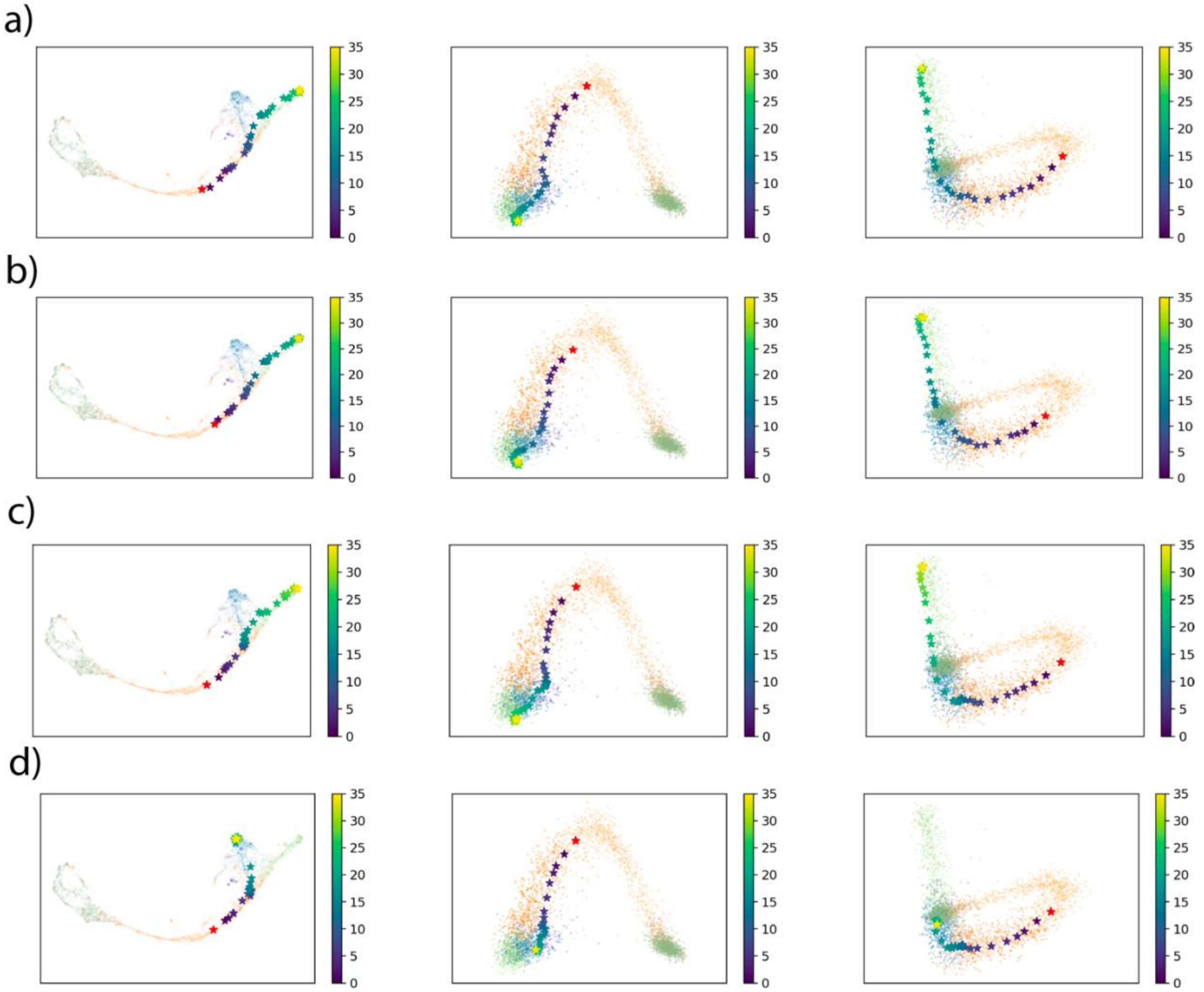

Fig. S1.

Example Pancreatic Endocrinogenesis Trajectories. a-c) Endocrine progenitor developing into pre-endocrine cells, then differentiating into beta cells. **d)** Alternative trajectory of progenitor cells developing into alpha cells.

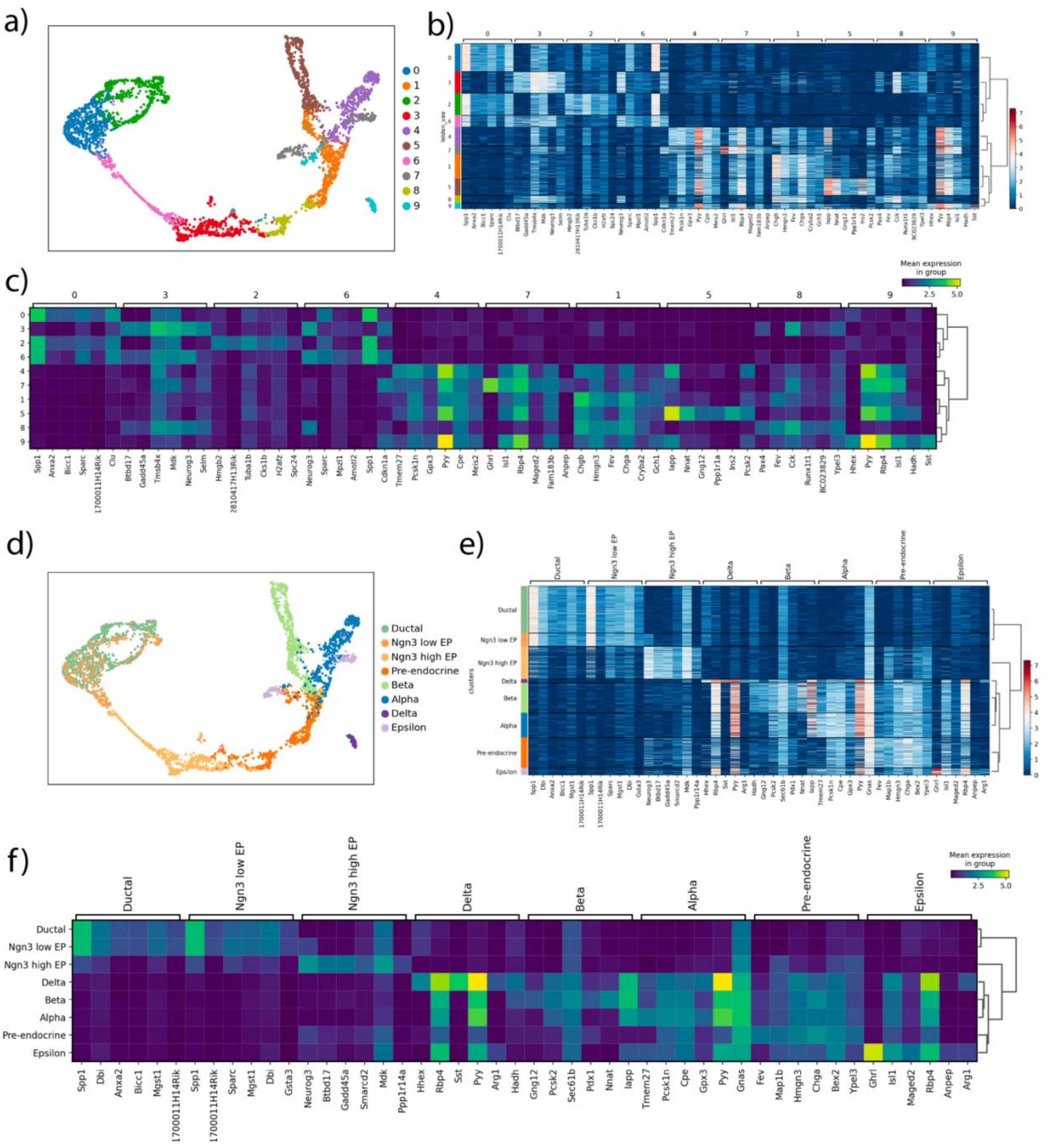

Fig. S2.

Differential Expression of Pancreatic Endocrinogenesis using VAE Latent Embeddings. a) Cells clustered in VAE embeddings using Leiden (with $K = 30$ nearest neighbors). The VAE embeddings are further projected into 2D for visualization with UMAP. **b-c)** Differentially expressed genes of cells clustered in VAE embeddings. **d)** Original cell labels in UMAP projected VAE embeddings. **e-f)** Differentially expressed genes of original cell labels.

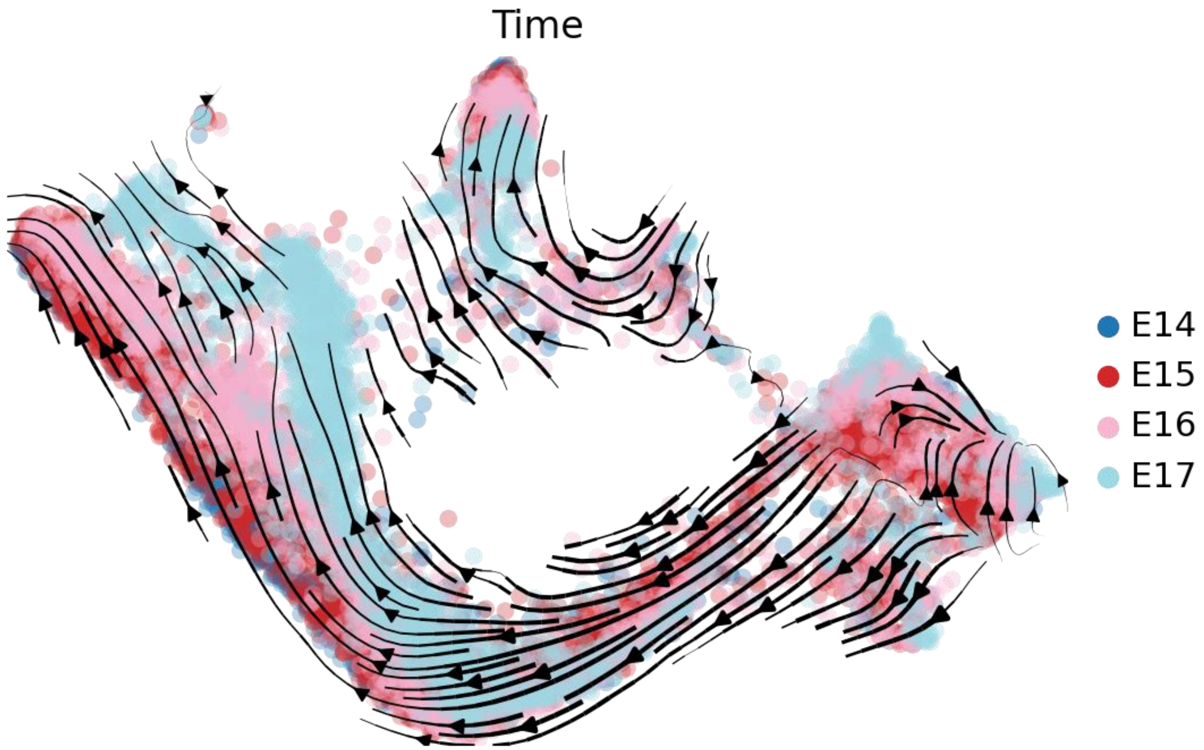

Fig. S3.

Batch Correction for Developing Mouse Neocortex. After filtering for the top 5,000 highly variable genes and normalizing within each time group, the "sc.pp.combat" function from *scanpy* was used to correct for batch effects. Further, the velocities were computed within each time group.

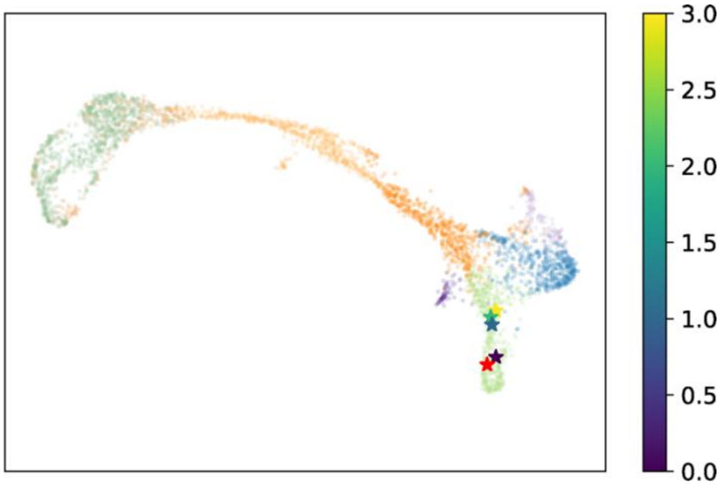

Fig. S4.

Example Pancreatic Endocrinogenesis Beta Cell Retrograde Trajectory. By integrating a matured beta cell reverse in time, we can generate retrograde trajectories to improve the correlation between gene modules. Here, the initial beta cell condition is in red, and the cell states tracing back in time are colored in viridis. Overall, the projection qualitatively shows that the retrograde trajectory lies within the span of the existing beta cell manifold.

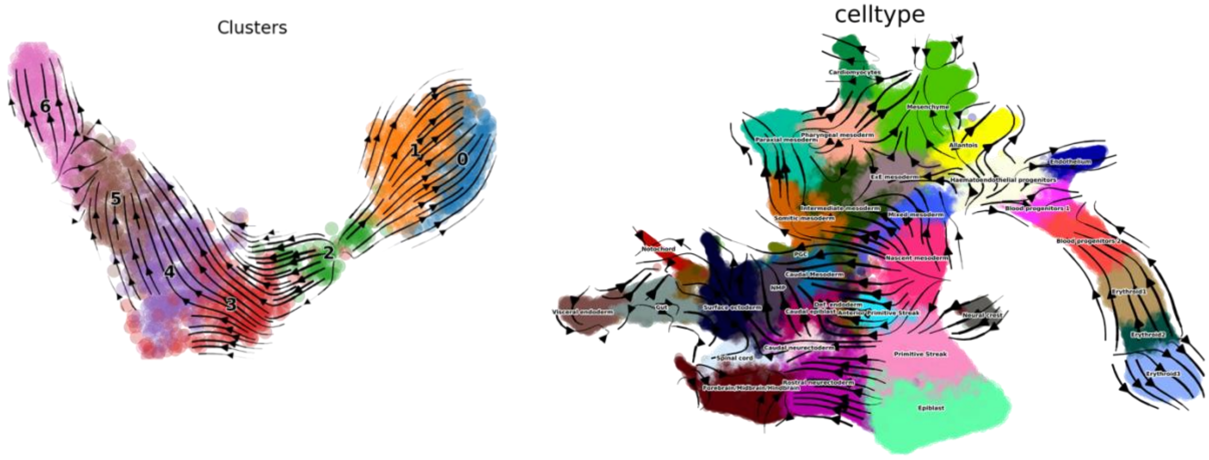

Fig. S5.

RNA Velocity Vector Field Visualization of the Human Forebrain and Mouse Gastrulation Datasets. The RNA velocity vector field of human forebrain (left) and mouse gastrulation (right) visualized in UMAP embedding colored by their cell types. These datasets were used for benchmarking the accuracy of velocity vector field representation learning in Figure 5c.

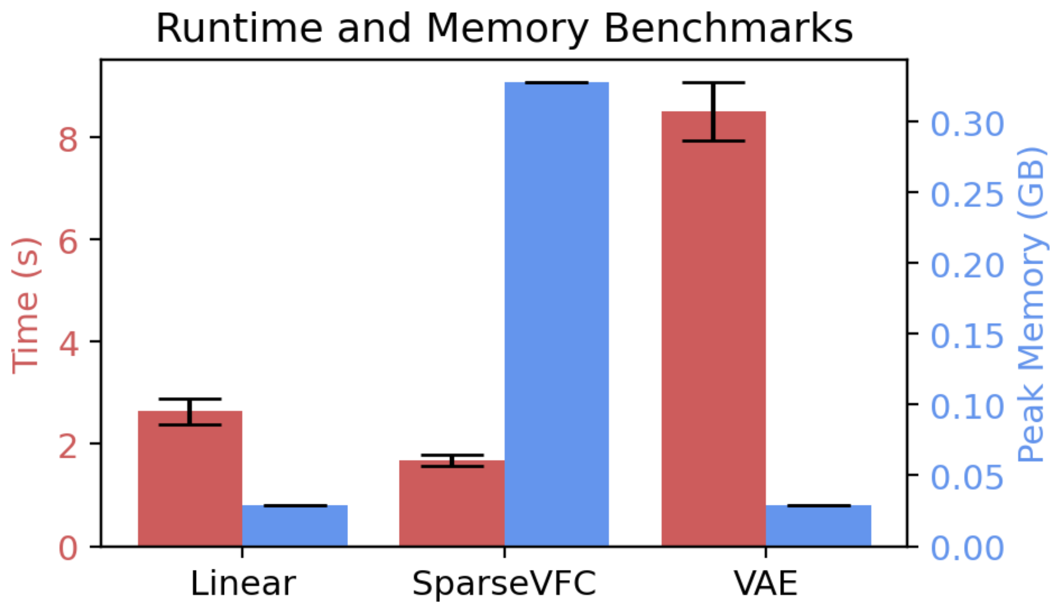

Fig. S6.

Runtime and Peak Memory Usage Comparisons Between Various Velocity Prediction Methods. Linear and VAE used similar amounts of peak memory since the model can be stored on the GPU, whereas SparseVFC used significantly more memory because it is a CPU-based model. In contrast, SparseVFC has the least runtime as a sparse approximation method, but VAE has the longest runtime due to the data exchanges between the CPU and the GPU.

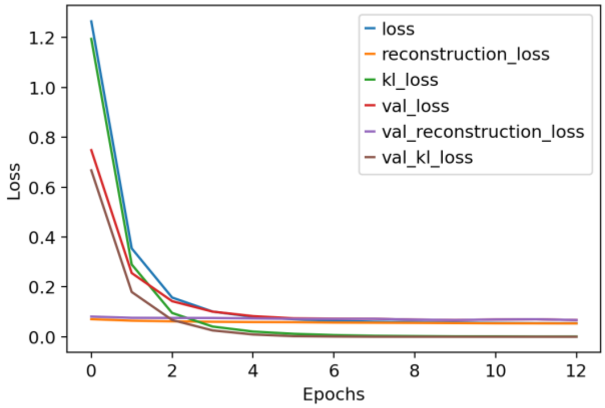

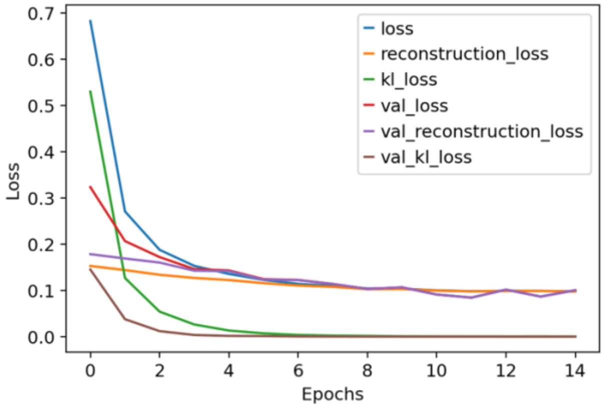

Fig. S7.

Variational Autoencoder Training. The training and validation loss (reconstruction, KL-divergence, and reconstruction with KL-divergence) of the variational autoencoder during training (left – pancreatic endocrinogenesis, right – dentate gyrus).

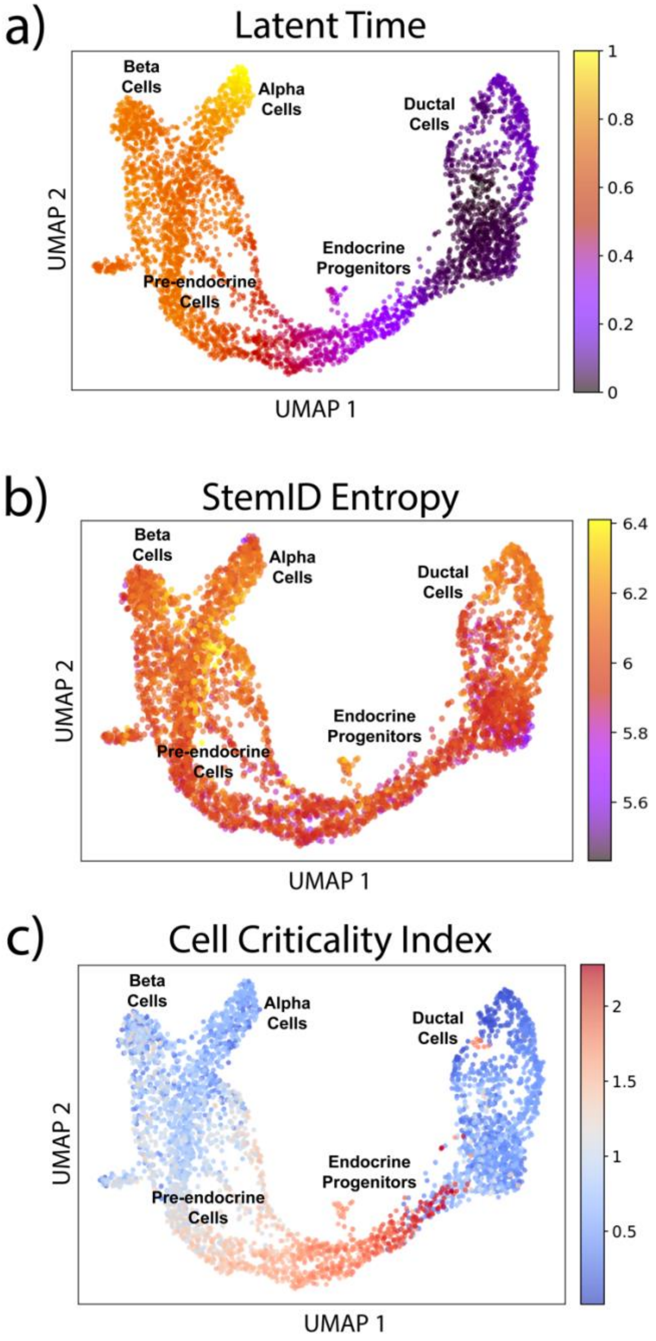

Fig. S8.

Comparing Velocity-based Latent Time, StemID Entropy, and Cell Criticality Index. In pancreatic endocrinogenesis, Velocity-based Latent Time provides a pseudotime temporal ordering of cells. StemID Entropy is designed to order the cells by pluripotency potential, but it fails here because the cells have similar amounts of uniformity in gene expression. Lastly, Cell Criticality Index highlights fixed points in the developmental landscape.

Gene Symbol	Gene Name	Function	Reference
**Smarca1**	SWI/SNF Related, Matrix Associated, Actin Dependent Regulator of Chromatin, Subfamily A, Member 1	Trans-differentiation from Beta to Alpha Cells	Kleiber, T., et al. (65).
**Arx**	Aristaless-related homeobox gene	Maintaining Alpha Cell Identity	Wilcox, Crystal L., et al. (66)
**Pam**	Peptidylglycine α-amidating Monooxygenase	Glucose Regulation	Thomsen, S.K., et al. (67).
**Bdnf**	Brain-derived neurotrophic factor	Modulating Glucagon Secretion	Hanyu, O, et al. (68)
**Irx2**	Iroquois Homeobox 2	Transcription Factors Shown to Contribute to Alpha Cell Fate Specification	Dorrell, C., et al. (69)

Table S1.

Alpha Cell Specific Driver Genes. Table of gene symbol, name, and the function of the alpha-cell-specific driver genes, with the corresponding reference.

Gene Symbol	Gene Name	Function	Reference
**Actr3b**	Actin-related protein 3B	ATP-binding Component and Mediates the Formation of Branched Actin Networks	Datta, Dibyadeep, et al. (70)
**Runx1t1**	Runt-related transcription factor 1 translocated to 1	Promote Neuronal Differentiation	Zou, Linqing, et al. (39)
**Slc7a5**	Solute Carrier Family 7 Member 5	Neurogenesis and Modulate Neuronal Electrophysiological Properties	Sokolov, Aidan M., Jennie C. Holmberg, and David M. Feliciano. (71)
**Neurod6**	Neuronal Differentiation 6	Neuronal Differentiation	Uittenbogaard, Martine, Kristin K. Baxter, and Anne Chiaramello. (72)
**Hmgcs1**	HMG–CoA Synthase 1	Cholesterol Homeostasis in Pyramidal Neurons	Valdez, Chris M., et al. (73)

Table S2.

Pyramidal Cell Specific Driver Genes. Table of gene symbol, name, and the function of the pyramidal-cell-specific driver genes, with the corresponding reference.

REFERENCES AND NOTES

1. F. Tang, C. Barbacioru, Y. Wang, E. Nordman, C. Lee, N. Xu, X. Wang, J. Bodeau, B. B. Tuch, A. Siddiqui, K. Lao, M. A. Surani, mRNA-seq whole-transcriptome analysis of a single cell. *Nat. Methods* **6**, 377–382 (2009).

2. S. Islam, U. Kjällquist, A. Moliner, P. Zajac, J.-B. Fan, P. Lönnerberg, S. Linnarsson, Characterization of the single-cell transcriptional landscape by highly multiplex RNA-seq. *Genome Res.* **21**, 1160–1167 (2011).

3. D. Ramsköld, S. Luo, Y.-C. Wang, R. Li, Q. Deng, O. R. Faridani, G. A. Daniels, I. Khrebtukova, J. F. Loring, L. C. Laurent, G. P. Schroth, R. Sandberg, Full-length mRNA-Seq from single-cell levels of RNA and individual circulating tumor cells. *Nat. Biotechnol.* **30**, 777–782 (2012).

4. X. Han, R. Wang, Y. Zhou, L. Fei, H. Sun, S. Lai, A. Saadatpour, Z. Zhou, H. Chen, F. Ye, D. Huang, Y. Xu, W. Huang, M. Jiang, X. Jiang, J. Mao, Y. Chen, C. Lu, J. Xie, Q. Fang, Y. Wang, R. Yue, T. Li, H. Huang, S. H. Orkin, G.-C. Yuan, M. Chen, G. Guo, Mapping the mouse cell atlas by microwell-seq. *Cell* **172**, 1091–1107.e17 (2018).

5. A. Saunders, E. Z. Macosko, A. Wysoker, M. Goldman, F. M. Krienen, H. de Rivera, E. Bien, M. Baum, L. Bortolin, S. Wang, A. Goeva, J. Nemesh, N. Kamitaki, S. Brumbaugh, D. Kulp, S. A. McCarroll, Molecular diversity and specializations among the cells of the adult mouse brain. *Cell* **174**, 1015–1030.e16 (2018).

6. A. Zeisel, H. Hochgerner, P. Lönnerberg, A. Johnsson, F. Memic, J. van der Zwan, M. Häring, E. Braun, L. E. Borm, G. L. Manno, S. Codeluppi, A. Furlan, K. Lee, N. Skene, K. D. Harris, J. Hjerling-Leffler, E. Arenas, P. Ernfors, U. Marklund, S. Linnarsson, Molecular architecture of the mouse nervous system. *Cell* **174**, 999–1014.e22 (2018).

7. P. V. Kharchenko, The triumphs and limitations of computational methods for scRNA-seq. *Nat. Methods* **18**, 723–732 (2021).

8. S. Linnarsson, S. A. Teichmann, Single-cell genomics: Coming of age. *Genome Biol.* **17**, 97 (2016).

9. P. He, B. A. Williams, D. Trout, G. K. Marinov, H. Amrhein, L. Berghella, S.-T. Goh, I. Plajzer-Frick, V. Afzal, L. A. Pennacchio, D. E. Dickel, A. Visel, B. Ren, R. C. Hardison, Y. Zhang, B. J. Wold, The changing mouse embryo transcriptome at whole tissue and single-cell resolution. *Nature* **583**, 760–767 (2020).

10. D. W. McKellar, L. D. Walter, L. T. Song, M. Mantri, M. F. Wang, I. De Vlaminck, B. D. Cosgrove, Large-scale integration of single-cell transcriptomic data captures transitional progenitor states in mouse skeletal muscle regeneration. *Commun. Biol.* **4**, 1–12 (2021).

11. F. Wu, J. Fan, Y. He, A. Xiong, J. Yu, Y. Li, Y. Zhang, W. Zhao, F. Zhou, W. Li, J. Zhang, X. Zhang, M. Qiao, G. Gao, S. Chen, X. Chen, X. Li, L. Hou, C. Wu, C. Su, S. Ren, M. Odenthal, R. Buettner, N. Fang, C. Zhou, Single-cell profiling of tumor heterogeneity and the microenvironment in advanced non-small cell lung cancer. *Nat. Commun.* **12**, 1–11 (2021).

12. A. Saadatpour, S. Lai, G. Guo, G.-C. Yuan, Single-cell analysis in cancer genomics. *Trends Genet.* **31**, 576–586 (2015).

13. C. Trapnell, D. Cacchiarelli, J. Grimsby, P. Pokharel, S. Li, M. Morse, N. J. Lennon, K. J. Livak, T. S. Mikkelsen, J. L. Rinn, The dynamics and regulators of cell fate decisions are revealed by pseudotemporal ordering of single cells. *Nat. Biotechnol.* **32**, 381–386 (2014).

14. M. Setty, V. Kiseliovas, J. Levine, A. Gayoso, L. Mazutis, D. Pe'er, Characterization of cell fate probabilities in single-cell data with Palantir. *Nat. Biotechnol.* **37**, 451–460 (2019).

15. X. Qiu, A. Rahimzamani, L. Wang, B. Ren, Q. Mao, T. Durham, J. L. McFaline-Figueroa, L. Saunders, C. Trapnell, S. Kannan, Inferring causal gene regulatory networks from coupled single-cell expression dynamics using scribe. *Cell Syst.* **10**, 265–274.e11 (2020).

16. G. L. Manno, R. Soldatov, A. Zeisel, E. Braun, H. Hochgerner, V. Petukhov, K. Lidschreiber, M. E. Kastriti, P. Lönnerberg, A. Furlan, J. Fan, L. E. Borm, Z. Liu, D. van Bruggen, J. Guo, X. He, R. Barker, E. Sundström, G. Castelo-Branco, P. Cramer, I. Adameyko, S. Linnarsson, P. V. Kharchenko, RNA velocity of single cells. *Nature* **560**, 494–498 (2018).

17. T. Stuart, A. Butler, P. Hoffman, C. Hafemeister, E. Papalexi, W. M. Mauck III, Y. Hao, M. Stoeckius, P. Smibert, R. Satija, Comprehensive integration of single-cell data. *Cell* **177**, 1888–1902.e21 (2019).

18. X. Han, Z. Zhou, L. Fei, H. Sun, R. Wang, Y. Chen, H. Chen, J. Wang, H. Tang, W. Ge, Y. Zhou, F. Ye, M. Jiang, J. Wu, Y. Xiao, X. Jia, T. Zhang, X. Ma, Q. Zhang, X. Bai, S. Lai, C. Yu, L. Zhu, R. Lin, Y. Gao, M. Wang, Y. Wu, J. Zhang, R. Zhan, S. Zhu, H. Hu, C. Wang, M. Chen, H. Huang, T. Liang, J. Chen, W. Wang, D. Zhang, G. Guo, Construction of a human cell landscape at single-cell level. *Nature* **581**, 303–309 (2020).

19. J. Cao, M. Spielmann, X. Qiu, X. Huang, D. M. Ibrahim, A. J. Hill, F. Zhang, S. Mundlos, L. Christiansen, F. J. Steemers, C. Trapnell, J. Shendure, The single-cell transcriptional landscape of mammalian organogenesis. *Nature* **566**, 496–502 (2019).

20. H. Matsumoto, H. Kiryu, C. Furusawa, M. S. Ko, S. B. Ko, N. Gouda, T. Hayashi, I. Nikaido, SCODE: An efficient regulatory network inference algorithm from single-cell RNA-seq during differentiation. *Bioinformatics* **33**, 2314–2321 (2017).

21. X. Qiu, Y. Zhang, J. D. Martin-Rufino, C. Weng, S. Hosseinzadeh, D. Yang, A. N. Pogson, M. Y. Hein, K. H. J. Min, L. Wang, E. I. Grody, M. J. Shurtleff, R. Yuan, S. Xu, Y. Ma, J. M. Replogle, E. S. Lander, S. Darmanis, I. Bahar, V. G. Sankaran, J. Xing, J. S. Weissman, Mapping transcriptomic vector fields of single cells. *Cell* **185**, 690–711.e45 (2022).

22. T. Clack, A. Shokry, M. Moffet, P. Liu, M. Faul, R. A. Sharrock, Obligate heterodimerization of Arabidopsis phytochromes C and E and interaction with the PIF3 basic helix-loop-helix transcription factor. *Plant Cell* **21**, 786–799 (2009).

23. G. A. Graf, L. Yu, W.-P. Li, R. Gerard, P. L. Tuma, J. C. Cohen, H. H. Hobbs, ABCG5 and ABCG8 are obligate heterodimers for protein trafficking and biliary cholesterol excretion. *J. Biol. Chem.* **278**, 48275–48282 (2003).

24. R. T. Chen, Y. Rubanova, J. Bettencourt, D. Duvenaud, Neural ordinary differential equations. *Adv. Neural Inf. Process. Syst.* **31**, 1–13 (2018).

25. S. L. Brunton, J. L. Proctor, J. N. Kutz, Discovering governing equations from data by sparse identification of nonlinear dynamical systems. *Proc. Natl. Acad. Sci. U.S.A.* **113**, 3932–3937 (2016).

26. A. Bastidas-Ponce, S. Tritschler, L. Dony, K. Scheibner, M. Tarquis-Medina, C. Salinno, S. Schirge, I. Burtscher, A. Böttcher, F. J. Theis, H. Lickert, M. Bakhti, Comprehensive single cell mRNA profiling reveals a detailed roadmap for pancreatic endocrinogenesis. *Development* **146**, dev173849 (2019).

27. H. Hochgerner, A. Zeisel, P. Lönnerberg, S. Linnarsson, Conserved properties of dentate gyrus neurogenesis across postnatal development revealed by single-cell RNA sequencing. *Nat. Neurosci.* **21**, 290–299 (2018).

28. D. J. Di Bella, E. Habibi, R. R. Stickels, G. Scalia, J. Brown, P. Yadollahpour, S. M. Yang, C. Abbate, T. Biancalani, E. Z. Macosko, F. Chen, A. Regev, P. Arlotta, Molecular logic of cellular diversification in the mouse cerebral cortex. *Nature* **595**, 554–559 (2021).

29. B. Pijuan-Sala, J. A. Griffiths, C. Guibentif, T. W. Hiscock, W. Jawaid, F. J. Calero-Nieto, C. Mulas, X. Ibarra-Soria, R. C. V. Tyser, D. L. L. Ho, W. Reik, S. Srinivas, B. D. Simons, J. Nichols, J. C. Marioni, B. Göttgens, A single-cell molecular map of mouse gastrulation and early organogenesis. *Nature* **566**, 490–495 (2019).

30. E. F. James, Bistability, bifurcations, and Waddington's epigenetic landscape. *Curr. Biol.* **22**, R458–R466 (2012).

31. V. Bergen, M. Lange, S. Peidli, F. A. Wolf, F. J. Theis, Generalizing RNA velocity to transient cell states through dynamical modeling. *Nat. Biotechnol.* **38**, 1408–1414 (2020).

32. M. E. Bechard, E. D. Bankaitis, S. B. Hipkens, A. Ustione, D. W. Piston, Y.-P. Yang, M. A. Magnuson, C. V. Wright, Precommitment low-level Neurog3 expression defines a long-lived mitotic endocrine-biased progenitor pool that drives production of endocrine-committed cells. *Genes Dev.* **30**, 1852–1865 (2016).

33. Z. Li, S. A. Langhans, Transcriptional regulators of Na, K-ATPase subunits. *Front. Cell Dev. Biol.* **3**, 66 (2015).

34. E. N. Lorenz, Deterministic nonperiodic flow. *J. Atmos. Sci.* **20**, 130–141 (1963).

35. M. R. DiGruccio, A. M. Mawla, C. J. Donaldson, G. M. Noguchi, J. Vaughan, C. Cowing-Zitron, T. van der Meulen, M. O. Huising, Comprehensive alpha, beta and delta cell transcriptomes reveal that ghrelin selectively activates delta cells and promotes somatostatin release from pancreatic islets. *Mol. Metab.* **5**, 449–458 (2016).

36. D. E. Wagner, A. M. Klein, Lineage tracing meets single-cell omics: Opportunities and challenges. *Nat. Rev. Genet.* **21**, 410–427 (2020).

37. Y. Gonda, H. Sakurai, Y. Hirata, H. Tabata, I. Ajioka, K. Nakajima, Expression profiles of insulin-like growth factor binding protein-like 1 in the developing mouse forebrain. *Gene Expr. Patterns* **7**, 431–440 (2007).

38. E. M. Batista, J. G. Doria, T. H. Ferreira-Vieira, J. Alves-Silva, S. S. Ferguson, F. A. Moreira, F. M. Ribeiro, Orchestrated activation of mGluR5 and CB 1 promotes neuroprotection. *Mol. Brain* **9**, 1–17 (2016).

39. L. Zou, H. Li, X. Han, J. Qin, G. Song, Runx1t1 promotes the neuronal differentiation in rat hippocampus. *Stem Cell Res. Ther.* **11**, 1–10 (2020).

40. S. G. Galfrè, F. Morandin, M. Pietrosanto, F. Cremisi, M. Helmer-Citterich, COTAN: ScRNA-seq data analysis based on gene co-expression. *NAR Genom. Bioinform.* **3**, lqab072 (2021).

41. D. Mercatelli, F. Ray, F. M. Giorgi, Pan-cancer and single-cell modeling of genomic alterations through gene expression. *Front. Genet.* **10**, 671 (2019).

42. B. D. Harris, M. Crow, S. Fischer, J. Gillis, Single-cell co-expression analysis reveals that transcriptional modules are shared across cell types in the brain. *Cell Syst.* **12**, 748–756.e3 (2021).

43. G. Karlebach, R. Shamir, Modelling and analysis of gene regulatory networks. *Nat. Rev. Mol. Cell Biol.* **9**, 770–780 (2008).

44. S. Li, P. Brazhnik, B. Sobral, J. J. Tyson, A quantitative study of the division cycle of Caulobacter crescentus stalked cells. *PLOS Comput. Biol.* **4**, e9 (2008).

45. H. H. McAdams, L. Shapiro, A bacterial cell-cycle regulatory network operating in time and space. *Science* **301**, 1874–1877 (2003).

46. J. Holtzendorff, D. Hung, P. Brende, A. Reisenauer, P. H. Viollier, H. H. McAdams, L. Shapiro, Oscillating global regulators control the genetic circuit driving a bacterial cell cycle. *Science* **304**, 983–987 (2004).

47. E. G. Biondi, S. J. Reisinger, J. M. Skerker, M. Arif, B. S. Perchuk, K. R. Ryan, M. T. Laub, Regulation of the bacterial cell cycle by an integrated genetic circuit. *Nature* **444**, 899–904 (2006).

48. M. S. Yeung, J. Tegnér, J. J. Collins, Reverse engineering gene networks using singular value decomposition and robust regression. *Proc. Natl. Acad. Sci. U.S.A.* **99**, 6163–6168 (2002).

49. D. C. Weaver, C. T. Workman, G. D. Stormo, Modeling regulatory networks with weight matrices, in *Biocomputing'99* (World Scientific, 1999), pp. 112–123.

50. L. Haghverdi, M. Büttner, F. A. Wolf, F. Buettner, F. J. Theis, Diffusion pseudotime robustly reconstructs lineage branching. *Nat. Methods* **13**, 845–848 (2016).

51. B. Sahu, T. Hartonen, P. Pihlajamaa, B. Wei, K. Dave, F. Zhu, E. Kaasinen, K. Lidschreiber, M. Lidschreiber, C. O. Daub, P. Cramer, T. Kivioja, J. Taipale, Sequence determinants of human gene regulatory elements. *Nat. Genet.* **54**, 283–294 (2022).

52. R. Andersson, A. Sandelin, Determinants of enhancer and promoter activities of regulatory elements. *Nat. Rev. Genet.* **21**, 71–87 (2020).

53. P. L. Ståhl, F. Salmén, S. Vickovic, A. Lundmark, J. F. Navarro, J. Magnusson, S. Giacomello, M. Asp, J. O. Westholm, M. Huss, A. Mollbrink, S. Linnarsson, S. Codeluppi, Å. Borg, F. Pontén, P. I. Costea, P. Sahlén, J. Mulder, O. Bergmann, J. Lundeberg, J. Frisén, Visualization and analysis of gene expression in tissue sections by spatial transcriptomics. *Science* **353**, 78–82 (2016).

54. D. B. Burkhardt, J. S. Stanley, A. Tong, A. L. Perdigoto, S. A. Gigante, K. C. Herold, G. Wolf, A. J. Giraldez, D. van Dijk, S. Krishnaswamy, Quantifying the effect of experimental perturbations at single-cell resolution. *Nat. Biotechnol.* **39**, 619–629 (2021).

55. M. Lotfollahi, F. A. Wolf, F. J. Theis, scGen predicts single-cell perturbation responses. *Nat. Methods* **16**, 715–721 (2019).

56. G. X. Y. Zheng, J. M. Terry, P. Belgrader, P. Ryvkin, Z. W. Bent, R. Wilson, S. B. Ziraldo, T. D. Wheeler, G. P. Mc Dermott, J. Zhu, M. T. Gregory, J. Shuga, L. Montesclaros, J. G. Underwood, D. A. Masquelier, S. Y. Nishimura, M. Schnall-Levin, P. W. Wyatt, C. M. Hindson, R. Bharadwaj, A. Wong, K. D. Ness, L. W. Beppu, H. J. Deeg, C. M. Farland, K. R. Loeb, W. J. Valente, N. G. Ericson, E. A. Stevens, J. P. Radich, T. S. Mikkelsen, B. J. Hindson, J. H. Bielas, Massively parallel digital transcriptional profiling of single cells. *Nat. Commun.* **8**, 1–12 (2017).

57. M. Abadi, TensorFlow: Learning functions at scale (2016); www.tensorflow.org/.

58. F. Chollet, Building autoencoders in keras. *Keras Blog* **14**, 98 (2016).

59. P. Virtanen, R. Gommers, T. E. Oliphant, M. Haberland, T. Reddy, D. Cournapeau, E. Burovski, P. Peterson, W. Weckesser, J. Bright, S. J. van der Walt, M. Brett, J. Wilson, K. J. Millman, N. Mayorov, A. R. J. Nelson, E. Jones, R. Kern, E. Larson, C J Carey, İ. Polat, Y. Feng, E. W. Moore, J. V. Plas, D. Laxalde, J. Perktold, R. Cimrman, I. Henriksen, E A Quintero, C. R. Harris, A. M. Archibald, A. H. Ribeiro, F. Pedregosa, P. van Mulbregt; SciPy 1.0 Contributors, SciPy 1.0: Fundamental algorithms for scientific computing in Python. *Nat. Methods* **17**, 261–272 (2020).

60. S. Beauregard, H. Haas, Pedestrian dead reckoning: A basis for personal positioning, in *Proceedings of the 3rd Workshop on Positioning, Navigation and Communication* (Shaker Verlag, 2006), pp. 27–35.

61. F. Pedregosa, G. Varoquaux, A. Gramfort, V. Michel, B. Thirion, O. Grisel, M. Blondel, P. Prettenhofer, R. Weiss, V. Dubourg, J. Vanderplas, A. Passos, D. Cournapeau, M. Brucher, M. Perrot, E. Duchesnay, Scikit-learn: Machine learning in Python. *J. Mach. Learn. Res.* **12**, 2825–2830 (2011).

62. D. Grün, M. J. Muraro, J.-C. Boisset, K. Wiebrands, A. Lyubimova, G. Dharmadhikari, M. van den Born, J. van Es, E. Jansen, H. Clevers, E. J. P. de Koning, A. van Oudenaarden, De novo prediction of stem cell identity using single-cell transcriptome data. *Cell Stem Cell* **19**, 266–277 (2016).

63. F. A. Wolf, P. Angerer, F. J. Theis, SCANPY: Large-scale single-cell gene expression data analysis. *Genome Biol.* **19**, 15 (2018).

64. D. V. Klopfenstein, L. Zhang, B. S. Pedersen, F. Ramírez, A. W. Vesztrocy, A. Naldi, C. J. Mungall, J. M. Yunes, O. Botvinnik, M. Weigel, W. Dampier, C. Dessimoz, P. Flick, H. Tang, GOATOOLS: A Python library for Gene Ontology analyses. *Sci. Rep.* **8**, 1–17 (2018).

65. T. Kleiber, G. Davidson, G. Mengus, I. Martianov, I. Davidson, Single cell transcriptomics reveal trans-differentiation of pancreatic beta cells following inactivation of the TFIID subunit Taf4. *Cell Death Dis.* **12**, 1–12 (2021).

66. C. L. Wilcox, N. A. Terry, E. R. Walp, R. A. Lee, C. L. May, Pancreatic α-cell specific deletion of mouse Arx leads to α-cell identity loss. *PLOS ONE* **8**, e66214 (2013).

67. S. K. Thomsen, A. Raimondo, B. Hastoy, S. Sengupta, X.-Q. Dai, A. Bautista, J. Censin, A. J. Payne, M. M. Umapathysivam, A. F. Spigelman, A. Barrett, C. J. Groves, N. L. Beer, J. E. M. Fox, M. I. McCarthy, A. Clark, A. Mahajan, P. Rorsman, P. E. MacDonald, A. L. Gloyn, Type 2 diabetes risk alleles in PAM impact insulin release from human pancreatic β-cells. *Nat. Genet.* **50**, 1122–1131 (2018).

68. O. Hanyu, K. Yamatani, T. Ikarashi, S. Soda, S. Maruyama, T. Kamimura, S. Kaneko, S. Hirayama, K. Suzuki, O. Nakagawa, H. Nawa, Y. Aizawa, Brain-derived neurotrophic factor modulates glucagon secretion from pancreatic alpha cells: Its contribution to glucose metabolism. *Diabetes Obes. Metab.* **5**, 27–37 (2003).

69. C. Dorrell, J. Schug, C. F. Lin, P. S. Canaday, A. J. Fox, O. Smirnova, R. Bonnah, P. R. Streeter, C. J. Stoeckert Jr., K. H. Kaestner, M. Grompe, Transcriptomes of the major human pancreatic cell types. *Diabetologia* **54**, 2832–2844 (2011).

70. D. Datta, D. Arion, K. M. Roman, D. W. Volk, D. A. Lewis, Altered expression of ARP2/3 complex signaling pathway genes in prefrontal layer 3 pyramidal cells in schizophrenia. *Am. J. Psychiatry* **174**, 163–171 (2017).

71. A. M. Sokolov, J. C. Holmberg, D. M. Feliciano, The amino acid transporter Slc7a5 regulates the mTOR pathway and is required for granule cell development. *Hum. Mol. Genet.* **29**, 3003–3013 (2020).

72. M. Uittenbogaard, K. K. Baxter, A. Chiaramello, NeuroD6 genomic signature bridging neuronal differentiation to survival via the molecular chaperone network. *J. Neurosci. Res.* **88**, 33–54 (2010).

73. C. M. Valdez, M. A. Smith, G. Perry, C. F. Phelix, F. Santamaria, Cholesterol homeostasis markers are localized to mouse hippocampal pyramidal and granule layers. *Hippocampus* **20**, 902–905 (2010).
